# Qualitative Analysis of Well-being Preparedness at an Emergency Medicine Residency Program

**DOI:** 10.5811/westjem.2018.10.39764

**Published:** 2018-11-26

**Authors:** David Diller, Jessica Osterman, Ramin Tabatabai

**Affiliations:** University of Southern California Keck School of Medicine, LAC+USC Medical Center, Department of Emergency Medicine, Los Angeles, California

## Abstract

**Introduction:**

There is significant variability in the preparedness of incoming interns at the start of residency training with regard to medical knowledge, procedural skills, and attitudes. Specialty-specific preparatory courses aimed at improving clinical skills exist; however, no preparatory courses targeting wellness promotion or burnout prevention have previously been described. Resident well-being has gained increasing attention from the Accreditation Council for Graduate Medical Education, and numerous studies have demonstrated high levels of burnout among resident physicians. The American Medical Association (AMA) divides resident well-being into the following six categories: nutrition, fitness, emotional health, financial health, preventative care, and mindset and behavioral adaptability. Using the AMA’s conceptual framework for well-being in residency, we performed a targeted needs assessment to support the development of a “pre-residency” well-being curriculum. Our aim was to discover what current residents and faculty felt were the perceived areas of under-preparedness, in relation to resident well-being, for incoming interns at the start of their residency training.

**Methods:**

Using a grounded theory approach, we conducted a series of semi-structured, focus group interviews. Focus groups consisted of junior residents (postgraduate years [PGY] 1-3), senior residents (PGY-4), and current faculty members. A standardized interview guide was used to prompt discussion and themes were identified from audio recording. We modified theories based on latent and manifest content analysis, and we performed member checking and an external audit to improve validity.

**Results:**

Participants noted variable exposure to both formal and informal well-being training prior to residency. Regardless, participants uniformly agreed that their past experiences did not adequately prepare them for the challenges, specific to burnout prevention, faced during residency training. Of the six domains of resident well-being described by the AMA, emotional health, mindset and behavioral adaptability, and financial health were the domains most cited for interns to be underprepared for at the start of residency training.

**Conclusion:**

Despite variability in prior medical school and life experiences, incoming interns were underprepared in several domains of well-being, including emotional health, mindset and behavioral adaptability, and financial health. Targeted interventions toward these areas of well-being should be piloted and studied further for their potential to mitigate effects of burnout among resident physicians.

## INTRODUCTION

There is significant variability in the preparedness of incoming interns at the start of residency training in terms of medical knowledge, technical skills, professional skills, and attitudes.^1^,^2^ While many specialty-specific, preparatory (boot camp) courses have been developed to increase intern confidence in their knowledge and skills, and to standardize a level of competence and performance prior to direct patient care, the authors are unaware of any published curricula targeting strategies to promote wellness, prevent burnout, and foster resilience.^3^,^4^

Resident well-being has gained increasing attention from the Accreditation Council for Graduate Medical Education (ACGME),^5^ and numerous studies have demonstrated high levels of burnout among resident physicians,^6^–^8^ with nearly 50% of incoming interns reporting burnout.^9^ The American Medical Association (AMA) states that resident well-being can be divided into the following six categories: nutrition; fitness; emotional health; financial health; preventative care; and mindset and behavioral adaptability.^10^ Little has been reported on emergency medicine (EM) resident preparedness in these domains, although there is evidence that the vast majority of EM residents receive no financial education in medical school or residency training.^11^ Using the AMA’s conceptual framework for well-being in residency, we performed a targeted needs assessment to support the development of a “pre-residency” well-being curriculum. The purpose of this study was to discover what current residents and faculty felt were the perceived areas of under-preparedness, in relation to resident well-being, for incoming interns at the start of their residency training.

## METHODS

Using a grounded theory approach,^12^,^13^ we conducted a series of semi-structured, focus group interviews in our EM residency program at the Los Angeles County + University of Southern California Medical Center, a large, urban, Level I trauma center. Focus group participation was voluntary, responses were kept confidential, and data were made anonymous. The University of Southern California Institutional Review Board approved the study under exempt status.

We used a convenience sample of junior residents (postgraduate years [PGY] 1-3), senior residents (PGY-4), and current faculty members to generate three focus groups. Recruitment was performed through a series of emails and announcements at didactic conference. The junior resident focus group consisted of one PGY-3 resident, one PGY-2 resident, and four PGY-1 residents. The senior resident focus group consisted of seven PGY-4 residents. The faculty focus group consisted of eight faculty members. Interview data were audio-recorded and collected in a private conference room within the hospital. The study authors each independently conducted a separate focus group using a standardized interview guide (Appendix A). To establish standardization and reliability across the three focus-group sessions, the authors met regularly to establish a homogenous approach. Additionally, after the principal investigator performed the initial focus group, the additional authors listened to the audio recording of the initial focus group prior to the recording of their individual focus-group sessions, to allow for further standardization. Only the respective interviewer and participants were present at each focus group.

**Population Health Research Capsule**

What do we already know about this issue?

*Little data exist regarding well-being preparedness among incoming emergency medicine interns*.

What was the research question?

What are the perceived areas of under-preparedness, related to resident well-being, for interns beginning residency?

What was the major finding of the study?

*Interns were underprepared in several domains of well-being, including emotional health, mindset and behavioral adaptability, and financial health*.

How does this improve population health?

*It provides a needs assessment for future pre-residency well-being curricula*.

As the study authors served as individual focus-group facilitators, it is important to note their individual qualifications, as focus group facilitation requires an important set of skills.^14^ All study authors serve as residency directors, with two of the authors (RT, DD) having completed medical education fellowships. Additionally, two of the authors (RT, DD) are currently enrolled in advanced degree programs in medical education and have taken courses specific to qualitative research methodology. The study authors all have professional interests in resident well-being research and curriculum development, and one of the authors (RT) has developed both institutional and specialty-specific national leadership roles within this domain. Although participants had knowledge about the researchers, they were unaware as to the reason the research was being conducted or the goals of the study. Finally, the primary author had specific interests in assessing the financial preparedness of residents, as this was the only domain within the AMA well-being conceptual framework that had previously-published content suggesting a need for improvement. With a recent increase in physician-specific, personal finance websites and books^15^ the primary author recognized the opportunity to use these resources as a component of the financial preparedness section of a “pre-residency” well-being curriculum. Accordingly, a series of questions within the interview guide specifically focused on the area of financial preparedness.

Non-verbatim transcription and coding was manually performed by the primary author directly from the audio recordings. Given the relatively small volume of focus-group content, the decision was made to forego the use of full-transcription software services, and rather focus on selective manual transcription and coding directly from the audio files, to better capture not only what interviewees were saying but also how they were saying it.^16^ To improve validity, audio recordings were listened to three times to ensure commentary was not omitted from the initial transcription and coding. Themes and sub-themes from both manifest and latent content analysis of the focus-group sessions were independently performed by each study author. Preliminary themes were consolidated by the primary author, and all three authors reviewed the consolidated document to ensure congruity to their independent analysis. We modified theories based on two rounds of content analysis and member checking to allow for codification and identification of common themes, which were agreed upon by all study authors through discussion and consensus (Appendix B).

## RESULTS

### Prior Experiences

Participants noted variable exposure to both formal and informal well-being training prior to residency training. Some participants received training during medical school, while others gained knowledge from prior life experiences. Training in medical school varied from longitudinal curricula that spanned the course of a semester to occasional lectures dispersed throughout the four years. Prior experiences that participants noted as preventative toward burnout included involvement in sports and regular exercise, adequate sleep and nutrition, yoga and meditative practices, and maintaining the activities or interests that formed one’s individual identity prior to residency training. Additionally, engagement through social interactivity, peer bonding, and mentorship were noted to be beneficial in promoting wellness and preventing burnout.

Regardless of past experiences, participants agreed that their past experiences did not adequately prepare them for the challenges specific to burnout prevention faced during residency training. For some, it was not a lack of knowledge regarding specific well-being domains (i.e., emotional health, financial health, nutrition, fitness), but the inability to apply their knowledge given the extrinsic challenges faced in residency training (e.g., clinical workload, financial pressures, psychological burden).

### Under-Preparedness in Well-being Domains

Of the six domains of resident well-being described by the AMA, emotional health, mindset and behavioral adaptability, and financial health were the domains most cited for interns to be underprepared for at the start of residency training. Participants noted that interns were unprepared to deal with the emotional exhaustion, the psychological burden of increased responsibility, and the ability to manage their self-doubt and feelings of imposter syndrome. In addition, interns were ill-equipped to manage their time effectively, both in terms of balancing clinical responsibilities with studying as well as achieving a healthy work-life balance. Participants felt that incoming interns were most knowledgeable with regard to fitness, nutrition, and preventative health, although the translation of this knowledge to practice was difficult given the time constraints and clinical workload of residency training.

The majority of participants acknowledged that incoming interns (and residents in general) had complete financial illiteracy in terms of their ability to manage debt, plan for retirement, budget, and invest. Opinions varied in how much financial illiteracy contributed to personal stress and burnout with one participant stating that it was “*the number one source of anxiety and frustration”* in his life, while another participant commented that of the aforementioned categories, her lack of financial understanding contributed the least to her “*emotional devastation*.”

### Variability in Responses Between Demographic Focus Groups

While common themes emerged from the content analysis, sub-themes existed between the three demographically selected focus groups (junior residents, senior residents, faculty). Junior residents commented on feelings of emotional exhaustion, difficulties with time management, and trouble dealing with both the psychological burden of increased clinical responsibility and feelings of self-doubt. Meanwhile senior residents noted difficulties with compassion fatigue, the cumulative psychological effects of having to constantly cope with tragedy, and their limited ability to self-identify symptoms of burnout. Faculty provided additional commentary, noting the psychological burdens of clinical documentation, the isolation from family and friends experienced during residency training, and the neglect of on-shift nutrition, all of which contribute to increased resident burnout.

## DISCUSSION

Incoming interns were underprepared for residency training with regard to several aspects of well-being including emotional health, financial health, and mindset and behavioral adaptability. Regardless of prior well-being training in medical school or past life experiences, focus group participants voiced both a need and a demand for a longitudinal well-being curriculum. We postulate that an opportune time to implement such a curriculum could be in the two-three month window between when medical students match into their chosen specialty and they begin their internship. This period of time has been described as having significant variability and questionable benefit to learners.^17^ With almost 50% of incoming interns experiencing some degree of burnout,^9^ intervention prior to residency training is prudent. Asynchronous, web-based, well-being curricula have previously been reported^18^–^21^ and would be an ideal modality for a well-being curriculum targeting incoming interns prior to the start of residency training.

The variability in responses between demographically separated focus groups suggests that the various elements of burnout affect physicians differently dependent on their level of training. Although this study was not designed to look at this phenomenon, intuitively it makes sense. As the most novice physicians in the emergency department, interns may inherently feel overwhelmed and emotionally exhausted. As residency progresses, feelings of emotional exhaustion and self-doubt may improve as more junior learners arrive, only to be replaced by compassion fatigue and depersonalization as the demanding training schedule progresses.

## LIMITATIONS

There were several notable limitations to this study. The study was conducted at a single residency program and limited additional demographic information was obtained from study participants, which limits its generalizability, although participants attended different medical schools and had a vast array of prior life experiences. We performed convenience sampling, as opposed to purposive sampling, due to limited group availability given clinical schedules. The sample size was relatively small, decreasing the statistical power of the study; additionally, the distribution of residents across focus groups (PGY 1-3 residents and PGY-4 residents as two separate groups) allowed for potential acquiescence bias within the former group. The groups were created in this manner to preserve equal numbers of focus-group participants across the study. However, the actual study population was relatively skewed towards PGY-4 residents and faculty, which also limits generalizability. Additionally, although we performed member checking, methods such as an external audit and triangulation would have been helpful to increase the rigor of the analysis.

By omitting software-facilitated verbatim transcription of audio recordings there was the potential for diminished capture and preservation of data fidelity as well as a reduction in the standardization of the data as displayed to the coders. However, prior research has questioned the necessity for verbatim data transcription in qualitative research,^22^,^23^ and the necessity and modality of data transcription remains an area of debate.

Regardless, this study’s results were promising. Future research should include a more robust sampling of interns/residents from different institutions and/or specialties to support the conclusions reached from this study. Additional research should include investigating targeted interventions to address well-being prior to interns commencing their residency training. A mixed-methods study using both validated well-being assessment metrics (e.g., wellness, burnout, resilience, mindfulness, etc.) alongside qualitative analysis in a multicenter or multi-specialty prospective study would be a preferred methodology to assess the efficacy of any piloted interventions. Further research should also be directed to assess the effect and influence of the institutional culture on residents’ well-being.

## CONCLUSION

Despite variability in prior medical school and life experiences, incoming interns were underprepared in several domains of well-being, including emotional health, mindset and behavioral adaptability, and financial health. Targeted interventions toward these areas of well-being should be piloted and studied further for their potential to mitigate effects of burnout among resident physicians.

## Supplementary Material




