# Supplementary material for: Qualitative Analysis of Well-being Preparedness at an Emergency Medicine Residency Program
Source: West J Emerg Med. 2018 Nov 26;20(1):122–6. doi: 10.5811/westjem.2018.10.39764 (PMC6324710; doi:10.5811/westjem.2018.10.39764)
Supplement: Supplementary file 1 [file wjem-20-122-s001.docx]

Interview Guide (Focus Groups - PGY 1-3; PGY 4; Attendings)

Welcome & Introduction

- Explain the rules to the focus group

*“Welcome and thank you for your willingness to participate. All of your responses will be audio recorded. Please speak clearly and one at a time. The idea is to have a stimulating discussion amongst yourselves. I will prompt or intervene only as needed. There are no right or wrong answers, only your opinions. So please, share your thoughts. As a final important reminder, your responses will remain anonymous. This focus group will last approximately 1 hour.” [2 minutes]*

Opening Question: (*Round Robin Question* - *Everyone answers, 10-20 seconds per answer*)

- Designed to identify characteristics that participants have in common
- Should be factual rather than opinion-based

*“Let’s start by going around the room: What medical school did you attend and what was the last job you held prior to becoming a doctor?” [3 minutes]*

Introductory Questions:

- To foster conversation between participants, not critical for analysis
- Opportunity for participants to reflect on past experiences and their connection with the overall topic

*“Great, now let’s open up to a more free-flowing discussion. What do you like best about this residency program?” [5 minutes]*

*“What are some of the most serious problems facing this residency program?” [5 minutes]*

Transition Questions:

- Help participants envision the topic on a broader scope
- Participants become aware of how others view the topic

*“Let’s talk about burnout in our emergency medicine residency program. What are the driving factors that lead to resident burnout in our program?” [5-10 minutes]*

*“Thinking about your medical school experience or any prior life experiences, what experiences helped you prepare for burnout prevention in your residency training?” [5-10 minutes]*

Key Questions: (*2-5 Questions*)

- Drive the study; require most attention in subsequent analysis

Uncued Questions → Cued Questions

*“What aspects of wellness and burnout prevention did you feel underprepared for as you entered residency training?” [5-10 minutes]*

[Cued Repsonse: After the group has answered the question, distribute the cue cards]

*“The American Medical Association has recognized these six categories as being key aspects in resident wellness training…Looking at this list, please elaborate on any categories that you feel have not received adequate training for prior to, or during your medical training.” [10-15 minutes]*

CUE CARD: [Distribute to each participant]

1. Nutrition (e.g., healthful food options and scheduled time to eat)
2. Fitness
3. Emotional Health
4. Preventive Care (e.g., dental care and provisions to see a primary care physician)
5. Financial Health (e.g., debt management, retirement planning & emergency fund support)
6. Mindset and Behavior Adaptability

[Cued Response (continued)]

*“Which categories would it be beneficial for residents to receive training on, prior to the start of their residency training?” [Prompt group to elaborate on responses] [10-15 minutes]*

*“Thank you. I want to turn our attention to financial health in particular. Financial health refers to debt management, retirement planning, emergency fund support, savings, budgeting, and wealth management.” [1 minute]*

*“How comfortable do you feel managing your own financial health?” [5 minutes]*

*“What training have you received up to now regarding managing your financial health?” [5 minutes]*

Ending Questions:

- Bring closure to the discussion
- Enable participants to reflect back on previous comments
  - All Things Considered Question
    - Participants state their final position on critical areas of concern

*“Ok. We’ve covered a lot and I appreciate your candor and participation. All things considered, what do you feel are the most important aspects to resident wellness that you would like to see incoming interns receive more training on?” [5 minutes]*

*“What advice do you have for us in creating such a training program?” [5 minutes]*

*“Any last comments?” [2 minutes]*

*“Ok, that concludes this focus group. Thank you* ***all for your participation****” [1 minute]*
